# Evidence of Interventions for the Prevention of Unintentional Injuries: Scoping Review

**DOI:** 10.2196/67877

**Published:** 2025-04-28

**Authors:** Sheela Shetty, Baby S Nayak, Anice George, Avinash Shetty, Vasudeva Guddattu

**Affiliations:** 1 Department of Child Health Nursing, Manipal College of Nursing, Manipal Academy of Higher Education, Madhava Nagar, Manipal, Udupi District, Karnataka, 576104, India, 91 9448174791; 2 Kasturba Hospital, Manipal Academy of Higher Education, Manipal, Udupi District, Karnataka, India; 3 Department of Data Science, Prasanna School of Public Health, Manipal Academy of Higher Education, Manipal, Udupi District, Karnataka, India

**Keywords:** prevention, injury, education, health, child, children, unintentional injury, disability, youth, surveillance, surveillance data, risk factor, injury intervention, literature search, scoping review, scoping literature review, policymaker, preventative measure, preventive measure

## Abstract

**Background:**

Unintentional injuries are the leading cause of death and disability among young children. Preventive strategies for unintentional injuries are mainly based on surveillance data and identifying risk factors.

**Objective:**

This study aimed to review and synthesize published literature that determined the effectiveness of interventions for preventing unintentional injuries among children.

**Methods:**

The methodological framework was supported by The Joanna Briggs Institute Reviewer’s Manual – Methodology for JBI Scoping Reviews as well as the PRISMA-ScR (Preferred Reporting Items for Systematic Reviews and Meta-Analyses extension for Scoping Reviews) guidelines. The inclusion criteria to include the studies in the review were unintentional injuries in children, interventions to prevent injuries, a brief description of the intervention and the outcome of the intervention, and articles published in a peer-reviewed journal and written in the English language.

**Results:**

In total, 21 articles were included in the review following the systematic search of key databases such as Web of Science, PubMed/MEDLINE, Scopus, ScienceDirect, and gray literature for studies published between July 2013 and May 2023. Of the 21 articles, 16 were randomized controlled trials, 4 were nonrandomized controlled trials, and 1 was a mixed method study. The findings of the review showed that interventions, either as a single measure (video-based teaching, testimonial story-based teaching, health education, storybook reading) or in combination (knowledge quiz and simulation test, module-based teaching along with personal counseling, and teaching with the help of video and poster), have shown a considerable decline in the number and severity of injuries. The studies included various target populations, including children and adolescents between 0 and 19 years old.

**Conclusions:**

The review results indicate the need to plan, implement, and reinforce preventive measures and techniques to reduce unintentional injuries among children. They can also serve as a useful indicator for policymakers.

## Introduction

### Background and Significance

Unintentional injuries among children are a major public health concern and result in significant childhood morbidity and mortality [1]. In addition to mortality risk, unintentional injuries can result in significant disability and disruption to quality of life [2,3]. Unfortunately, over 95% of all child injury deaths occur in low- and middle-income countries, resulting in a highly inequitable burden [4]. In India alone, unintentional injuries contribute to 9.1 deaths per 100,000 population, while transport injuries account for 2.8 deaths per 100,000 population [5].

Childhood encompasses different stages of emotional, physical, and brain development, ranging from newborn to adolescence. The age between birth and 5 years is a time of rapid changes in children’s physical and cognitive abilities that can increase their risk of unintentional injury [6]. Temperamental attributes such as increased activity level and impulsiveness in young children have been associated with proneness to injury [7]. Each of the new developmental stages in children brings changes in their physical, cognitive, or social development and the emergence of new injury hazards, thus emphasizing the importance of awareness of how injuries occur in certain populations to anticipate and avoid the risks [8].

Unintentional injuries were noted among children of younger mothers, overactive children, children belonging to extended or joint families, and children from urban dwellings, which assists in the identification of contributing risk factors to formulate strategies aimed at risk reduction and prevention of childhood injuries [9]. The environmental risk assessment found unsafe electrical points, unsafe stairs, unsafe kitchens with access to sharps, access to active fire, and unsafe furniture and objects as the most common risk factors leading to home injuries among young children [10]. Children left unsupervised or inadequately supervised may sustain negative physical, mental, or social outcomes [11].

The most challenging aspect for caregivers is to provide a safe environment for children to minimize injuries at home [12]. Toddlers and preschoolers are fragile as per their physical development and they spend most of their time at home compared to school-age children. This might impose a higher risk of developing unintentional injuries among children of this age [13]. The development of effective home hazard reduction educational materials could have a significant impact on the burden of home injuries in children, particularly in lower-income countries [14].

Studies are available on interventions to prevent unintentional injuries that are effective in improving various parameters such as knowledge, attitude, practice, and home environment.

### Objectives

This scoping review aimed to review and synthesize published literature that determined the effectiveness of interventions for preventing unintentional injuries among children and the magnitude of the outcomes to support children and families. Additionally, it aims to provide an overview of the various interventions and understand those that are effective in preventing unintentional injuries. The results of this review can assist researchers and health care professionals in implementing injury prevention interventions and creating awareness among caregivers. The search question guiding this scoping review study was: what evidence exists that determines the implementation and effectiveness of interventions to prevent unintentional injuries in children? The review also focused on the most cost-effective interventions for the prevention of unintentional injuries for implementation in resource-limited settings.

## Methods

### Overview

This scoping review examined the relevant literature on interventions addressing unintentional injuries among children. The methodological framework was supported by the Joanna Briggs Institute Reviewer’s Manual – Methodology for JBI Scoping Reviews, which builds on the framework introduced by Arksey and O’Malley [15] of Population, Concept, and Context, to create our search terms, with the population being parents or caregivers of children aged <12 years and children with age ranging from 0 to 19 years. The core concept is the prevention of unintentional injuries and includes interventions designed within the context of home, community, well-baby clinic, and preschool settings.

The review process followed 5 stages as outlines by Arksey and O’Malley [15]: stage 1, identifying the research question; stage 2, identifying relevant studies; stage 3, selecting studies; stage 4, charting the data; and stage 5, collating, summarizing, and reporting the results. The optional stage 6 is a consultation exercise to inform and validate the review findings with critical stakeholders, which was not carried out in this review. The methodological rigor of the individual studies was not assessed. However, each study was assessed for importance and coherence with the review question. We used the PRISMA-ScR (Preferred Reporting Items for Systematic Reviews and Meta-Analyses Extension for Scoping Reviews) guidelines to enhance transparency in our approach to the scoping review study [16]. The PRISMA-ScR guidelines checklist is depicted in Checklist 1.

### Stage 1: Identifying the Research Question

The review question was as follows: what evidence exists that determines the implementation and effectiveness of interventions to prevent unintentional injuries in children? The objectives of the review were (1) to provide a comprehensive overview of interventions to prevent unintentional injuries at home and (2) to identify the impact of interventions and outcomes on reducing the occurrence/incidence of unintentional injuries and improving the knowledge of parents/families regarding preventive strategies. The keywords used for the search were “injuries,” “unintentional injuries,” “children,” “prevention,” and “interventions.”

### Stage 2: Identifying Relevant Studies

The literature research included studies published between July 2013 and May 2023. Two independent reviewers (BSN and SS) searched the MEDLINE/PubMed, Scopus, ProQuest, Embase, and CINAHL databases for relevance. Search strategies specific to each database were developed. Any discrepancy in the extracted data was again rechecked by a third reviewer (AG) to resolve the discrepancy. AS provided subject expertise, and VG provided statistical input. The free-text terms and controlled vocabulary terms were combined via the relevant Boolean operators—AND, OR, and NOT. The bibliography and citations of the included studies were further searched to identify any additional studies pertinent to the review.

### Stage 3: Selecting Studies

The articles identified through searches were exported to Rayyan software for duplicate removal and title and abstract screening. Concerns with data extraction were resolved in consultation with the team members. Based on the eligibility criteria, relevant articles were reviewed and selected.

We included peer-reviewed, free, full-text articles published in the English language. Narrative reviews, critical reviews, systematic reviews, and meta-analyses, as well as nonempiric publications like editorials, opinion pieces, case reports, conference abstracts, and commentaries, were excluded. Studies that reported any interventions targeting a reduction or prevention of the incidence/occurrence of unintentional injuries among children or improved knowledge or change in behavior of parents were included. Quantitative interventional studies such as randomized controlled trials (RCT), non-RCT studies, quasi-experimental studies, before-and-after design, crossover designs, and mixed methods studies, which include interventions, were included. Observational studies and qualitative studies were excluded from the scope of this review.

### Stage 4: Charting the Data

The data extraction was carried out by 2 reviewers (BSN and SS), and any discrepancy in the extracted data was again rechecked by a third reviewer (AG). Data from the articles were extracted into an extraction table. The table included columns for authors, country of origin, year of publication, objectives, research design, sample size, target population, details of intervention, time frame of data collection, and main findings. Pilot testing of the data extraction framework was performed on 3 studies that were reviewed before the data extraction. No modifications were carried out in the data extraction framework. The summary of data extraction is depicted in Table 1.

**Table 1.** Description of data gathering measures of selected studies.

Author, year, country	Objective and study design	Target population	Intervention details	Measures and data collection	Findings and outcomes
Feng et al [17], 2023, China	To explore the mechanisms by which online social-network–based health education is effective in reducing unintentional injuries. RCT^a^.	Parents of children aged 0-3 years (n=138)	Health education intervention to improve knowledge, skill, and behavior of parents regarding unintentional injuries through a WeChat account for 12 weeks, and communication of parents with each other and community childcare doctor for 9 months.	The knowledge, skills, and beliefs of parents regarding unintentional injuries in children were assessed before and after the intervention.	Of 76 parents in the intervention group, 49 (64.5%) demonstrated better understanding and effective communication compared to 11 (31.9%) parents of 66 in the control group. A positive impact on enhancing parents’ knowledge, skills, and beliefs about unintentional childhood injuries was noted.
Ning et al [18], 2019, China	To assess the effectiveness of an app-based intervention to prevent unintentional injury. RCT.	Caregivers of preschool children aged 3-6 years (n=1980)	An app-based parenting education on unintentional injury prevention, with submodules to support interaction among users, surveys, and feedback.	Assessment of injury incidence, caregiver’s attitude toward injury prevention, and safety behaviors measured at baseline and at 3- and 6-month follow-up visits.	No change in the incidence of injury and caregiver’s attitude in either of the groups noted during the 6-month follow-up. Changes in injury prevention behavior were greater in the intervention group (B=.87, 95% CI 0.33‐1.42).
Feng et al [19], 2022, China	To evaluate the effectiveness of an online social community–based parental health education intervention in preventing unintentional injuries in children between 0 and 3 years of age. RCT.	Parents of children aged 0-3 years (n=365)	WeChat Group and WeChat official account named, ”Children Safety and Health.” Thirty studies and 30 text messages on unintentional injury prevention and videos on first aid measures were produced and sent to the parents’ WeChat group (intervention group).	Beliefs about unintentional injuries, skill in first aid measures for injuries, and behavior toward unintentional injuries were measured.	A significant difference in the occurrence of injuries was observed between the intervention and control groups (OR^b^ 1.71, 95% CI 1.02‐2.87; *P*=.04). The skill component showed a significant difference (*P*=.06) in the area of first aid for a tracheal foreign body.
Shen et al [20], 2016, China	To evaluate the efficacy of a testimonial-based video intervention in reducing drowning risk among school-aged children. RCT.	Children in third and fourth grade (n=280)	Testimonial-based intervention on drowning prevention; a 36-minute video of 4 testimonial stories about actual near-drowning experiences.	Safety knowledge on prevention of drowning, child-perceived vulnerability, and child-simulated behavior in and around the water measured at baseline and a week after the intervention.	A significant improvement was observed in children’s safety knowledge of drowning risk (*F*_1,250_^c^=7.04; *P*=.008) and safe, simulated behaviors (*F*_1, 245_=8.27; *P*=.004) related to playing in and near water. A minimal impact on children’s perceived vulnerability to drowning risk was found.
Wang et al [21], 2018, United States	To evaluate the effectiveness of an intervention grounded in social cognitive theory on the reduction of home safety problems. RCT.	Mother-toddler (12‐32 months) dyad (n=277)	Safety intervention through health education, goal setting, and social support on fire and fall prevention, poison control, and car seat use.	Assessment of home safety problems and self-efficacy behavior measured at baseline and 6 and 12 months after the intervention.	There were fewer safety problems in the intervention group (between-group difference in change over time *β*=−0.54, 95% CI −0.05 to −1.03; *P*=.035) and decreased self-efficacy scores were noted at 12 months follow-up.
Weaver, et al [22], 2019, United States	To evaluate the effectiveness of a tailored parenting program on parenting behaviors. RCT.	Caregivers of children between 0 and 5 years (n=125)	RISE UP, a tailored parenting program to promote nurturing, child development, resiliency, social connection, and support within the context of unintentional injury prevention.	Safety behaviors of parents related to unintentional injury areas were assessed.	Postintervention follow-up showed a decrease in parenting injury risk scores (95% CI 25-42) and the intervention promoted positive parenting behaviors.
Swartz et al [23], 2013, United States	To evaluate the effectiveness of Keeping Baby Safe In and Around the Car, a video-based DVD on child safety seat installation and use. RCT.	Parent or custodian of a child aged 0-24 months (n=195)	Self-directed viewing of Keeping Baby Safe In and Around the Car, a video-based DVD lasting 45 minutes	Pre- and posttest assessment of vehicle safety knowledge quiz and child safety seat installation simulation test.	A significant improvement in parents’ knowledge (*F*-value 103.71; *P*<.001) of car seats and their ability to discriminate the critical elements of correct car seat installation was found among the intervention group.
McKenzie, et al [24], 2021, United States	To evaluate the effect of a mobile technology–based health behavior change intervention on parental safety knowledge and behavior in preventing unintentional injuries. RCT.	Parents and caregivers of children aged between 0 and 12 years (n=5032)	The Make Safe Happen App was developed to help parents and caregivers learn about making their homes safer for children. It includes room-by-room safety checklists and links to purchase home safety products from Amazon.com.	Safety knowledge and safety actions of parents/caregivers were assessed during the pretest and posttest.	Mean knowledge score significantly increased between the pretest and posttest: intervention (8.45‐10.32; *P*<.0001) and control participants (8.51‐8.87; *P*=.0064). There was an increase in the percentage of participants who reported doing all repeated safety actions from 71.1% (pretest) to 77.3% (posttest) for the intervention group (*P*=.0001).
Banerjee et al [25], 2021, India	To evaluate the effectiveness of the child-to-child approach in preventing unintentional injuries in children. Non-RCT.	Children and adolescents from 0-19 years (n=397)	Older adolescents of the family were trained in first aid and CPR^d^, road safety, traffic rules, injury prevention, and immediate care. They were made to disseminate the knowledge to their younger siblings and other family members.	The magnitude of injuries, time taken for recovery from injuries, knowledge of participants, and practice of the family were assessed during the pre- and postintervention period.	The postintervention measurement showed a significant reduction in the incidence of injuries (intervention: 16; 2.03, 0.06‐4.0; control: 29; 3.62, 1.03‐6.2; *P*<.001) and improvement in knowledge and practice on injuries in the intervention group. The total time taken for recovery was also evident in the intervention group (143 vs 95 days).
George et al [26], 2021, India	To examine the effect of a home safety supervisory program on childhood safety, self-reported home hazards, and caregivers’ supervisory attitude. RCT.	Caregivers of children aged between 2-5 years (n=130)	The Home Safety Supervisory Program consisted of a video and poster on “safe home, safe child” and individual home visits.	Injury patterns, home safety practices, and self-reported home hazard practices were assessed at baseline and 1 month after the intervention.	Significant difference in the mean baseline scores of caregivers’ self-reported home hazard practices between the 2 groups (*P*<.001) and an improvement in the supervisory attitudes of caregivers in the intervention group (*P*<.001) were observed.
Holla et al [27], 2021, India	To evaluate the effectiveness of school-based interventions in promoting child safety and reducing unintentional injuries. RCT.	Children from classes 5 to 7 (n=1100)	A comprehensive pictorial child safety and injury prevention module was taught by 2 teachers on a regular basis (25‐30 hours on average/school)	Incidences and types of unintentional injuries were assessed during the pretest and at follow-up visits at 3, 6, and 10 months after the intervention.	The incidence of unintentional injuries declined from baseline until the end of the study in the intervention and control groups. However, the decline in incidence was higher in the intervention group (50.4% vs 12.7%; *P*<.001).
Taylor et al [28], 2023, United Kingdom	To assess the effectiveness of systematically delivered, evidence-based home safety promotion for improving child home-safety practices. Non-RCT.	Families consisting of parents or caregivers of children between the ages of 2 and 7 months (n=361)	Stay One Step Ahead, a multievidence-based intervention for families, consisted of monthly safety messages through quizzes, posters, and flyers; home safety activities; home safety checklists; and educational safety weeks for families on common injuries.	Home safety outcomes measures were assessed at baseline and 12 and 24 months of follow-up.	The families in the intervention group showed significantly more improvement in storage of poisons out of reach (OR 1.81, 95% CI 1.06-3.07; *P*=.029). The total number of home safety measures used by the intervention families was significantly more than the control group families at 12 months (β=0.34, 95% 0.06-0.63; *P*=.019) and 24 months follow-up (β=0.46, 95% CI 0.13-0.79*; P*=.006).
Cowley et al [29], 2021, United Kingdom	To assess the impact of SafeTea on the knowledge and behavior of parents with regard to the prevention of scalds and first aid for burns. Mixed method design.	Parents or caregivers of children less than 5 years of age	SafeTea, a community-based intervention that provides information to parents regarding the risk factors for hot drink scalds and first aid for burns. The campaign included video clips, posters, leaflets, and charts distributed to schools, nurseries, and parent groups.	Knowledge and behavior of parents related to the prevention of scalds and first aid for burns were assessed.	Qualitative analysis under 4 themes: “reach,” ”engagement“, ”acceptability,” and ”impact/behavioural change.” Reach and engagement declined after the first month due to decreased publicity and social media promotion. Changes in parents’ behavior to minimize the risk of burns noted. Awareness of parents on the dangers of hot drinks and use of appropriate first aid measures improved.
Morrongiello et al [30], 2013, Canada	To evaluate the efficacy of the Supervising for Home Safety program on mothers’ supervision practices. RCT.	Caregivers of children between 2 and 5 years of age (n=186)	Watchful Parents, Safe Children video (20 minutes), followed by a structured discussion for 40 minutes. Mothers were given supervision diary recording forms for an 8-week baseline period.	Two time points of diary-based recording of postintervention practices—after 1 month and 3 months.	Significant decrease in time the children were unsupervised (*F*_1,83_=4.81; *P*=.04), higher levels of supervision (*t*_166_=2.99; *P*<.01), and an increase in attention to the child, immediately (*t*_67_=5.22; *P*<.01) and 3 months (*t*_67_=3.84; *P*<.01) after the intervention.
Morrongiello et al [31], 2021, Canada	To evaluate the effectiveness of a storybook about home safety on preschoolers’ safety knowledge and injury risk behaviors. RCT.	Preschool children aged 3.5 to 5.5 years (n=59)	A storybook, “Careful Puppy Saves the Day,“ with information on different hazards in and around the home. Mothers were instructed to read the book with their children for 10 min/day, 4 times/week.	Knowledge about hazards and injury risk behavior was assessed at baseline and 4 weeks after the intervention.	Children in the intervention group were able to identify more hazards, showed fewer risky behaviors, and provided a more comprehensive explanation of safety (ηp2=0.13, 0.19, and 0.51, respectively).
Khan et al [32], 2023, Pakistan	To evaluate the effectiveness of the long-term effect of 2 injury prevention interventions on the prevalence of home injury hazards. Non-RCT.	Caregivers of children under the age of 5 years (n=312)	Two interventions were included: an educational pamphlet and a tutorial. Both consisted of information on injury hazards for children that are commonly found in homes along with the strategies to reduce or eliminate those hazards.	Reduction in home injury hazards was classified into 6 types: falls, burns, poisoning, drowning, cut injuries, and choking, which were assessed at baseline, 3 months after the intervention, and 2 years after the intervention.	The long-term outcome showed a significant reduction in injury hazards in the tutorial group when compared to the educational pamphlet group: falls (IRR^e^ 0.24, 95% CI 0.08‐0.71), drowning (IRR 0.45, 95% CI 0.85‐0.98), burns (IRR 0.56, 95% CI 0.33‐0.78), poisoning (IRR 0.53, 95% CI 0.44‐0.77), and breakable objects within reach of child (IRR 0.62, 95% CI 0.39‐0.99).
Tajiki et al [33], 2022, Pakistan	To examine the efficacy of a training program based on the health belief model in burn prevention knowledge in mothers of children aged 1-3 years. RCT.	Mothers of children aged between 1 and 3 years (n=64)	The educational intervention on preventing burns was delivered using lectures and PowerPoint slides, educational pamphlets, and illustrated books. It was a 45-minute session per week for a period of 6 weeks.	The questionnaire on the Health Belief Model for the prevention of burns in children was administered before the intervention, immediately after the intervention, and 2 months after the intervention.	There was a significant difference between the groups in terms of perceived susceptibility and severity during immediate and 2 months postintervention (control: 11.9, SD 0.53 vs 13.34, SD 0.82; intervention: 18.06, SD 0.24 vs 15.93, SD 0.24*; P*<.001). Significant difference in mean scores of perceived barriers and benefits (control: 9.78, SD 0.49; 9, SD 0; *P*=.6; intervention: 11, SD 0; 11.5, SD 1.04; *P*<.001).
Choi and Ahn [34], 2021, Republic of Korea	To evaluate the effectiveness of mobile-based parental education programs in preventing unintentional childhood injuries. RCT.	Parents or relatives caring for infants and toddlers (n=167)	Two groups. First group: e-learning program for preventing unintentional injuries. Second group: electronic document distribution group, which received a PDF of e-learning content.	Safety knowledge and safety behavior regarding unintentional injuries were assessed during the pretest and 2 weeks after the intervention.	There was no significant difference found in the safety knowledge of the participants in both intervention groups. However, the safety behavior between the 3 groups showed statistically significant differences (*F*=10.09; *P*<.001).
van Beelen et al [35], 2014, the Netherlands	To evaluate the effectiveness of web-based, tailored safety information combined with personal counseling on parents’ safety behaviors. RCT.	Parents with a child aged 5-8 months (n=1292)	E-health4Uth module, along with personal counseling on safety in and around the home on prevention of falls, poisoning, drowning, and burns in children aged between 12 and 24 months.	Parents’ child safety behavior on prevention of falls, poisoning, drowning, and burns was assessed at baseline and 6 months after the intervention.	Parents in the intervention group showed significantly less unsafe behavior when compared with control group parents. Parents positively rated the E-health4Uth home safety intervention as an effective source of information (mean 4.05, SD 0.62).
Myers et al [36], 2022, Israel	To evaluate the effect of the intervention on injuries in children aged 0-4 years. Non-RCT.	Children aged 0-4 years and 5-17 years through home visits (n=6334)	The multifaceted intervention included a youth leadership program, workshops in well-baby clinics and preschools, home visits, and a media campaign.	Data on visits to the emergency room and hospitalization were obtained from the hospital records during the pre- and postintervention periods.	There was a significant reduction in emergency room visits (7.6%) in children between 0 and 3 years during postintervention period. Admissions to the hospital for burns and falls reduced. A greater reduction in emergency room visits (*P*=.038; *P*=.004) was observed for children aged 0-4 years in towns that started the intervention earlier.
Cheraghi et al [37], 2014, Iran	To assess the effect of the Health Belief Model on the education of mothers for promoting safety and preventing injury. RCT.	Mothers with at least 1 child below the age of 5 years (n=120)	Four sessions, 1 hour each, twice per week, on factors affecting mothers’ knowledge and practices and Health Belief Model constructs.	Assessment of mother’s knowledge, practice, Health Belief Model constructs, and injury history before and 2 months postintervention.	There was a significant mean difference in mothers’ knowledge (3.98), practice (2.47), and Health Belief Model constructs in the intervention group (*P*<.001). The number of injuries decreased from 7 to 2 in the intervention group.

a RCT: randomized controlled trial.

b OR: odds ratio.

c *F*: analysis of variance.

d CPR: cardiopulmonary resuscitation.

e IRR: incidence risk ratio.

### Stage 5: Collating, Summarizing, and Reporting the Results

Data extracted from the included studies were collated and textually summarized and findings were tabulated as key categories.

## Results

### Overview

**Figure 1.**
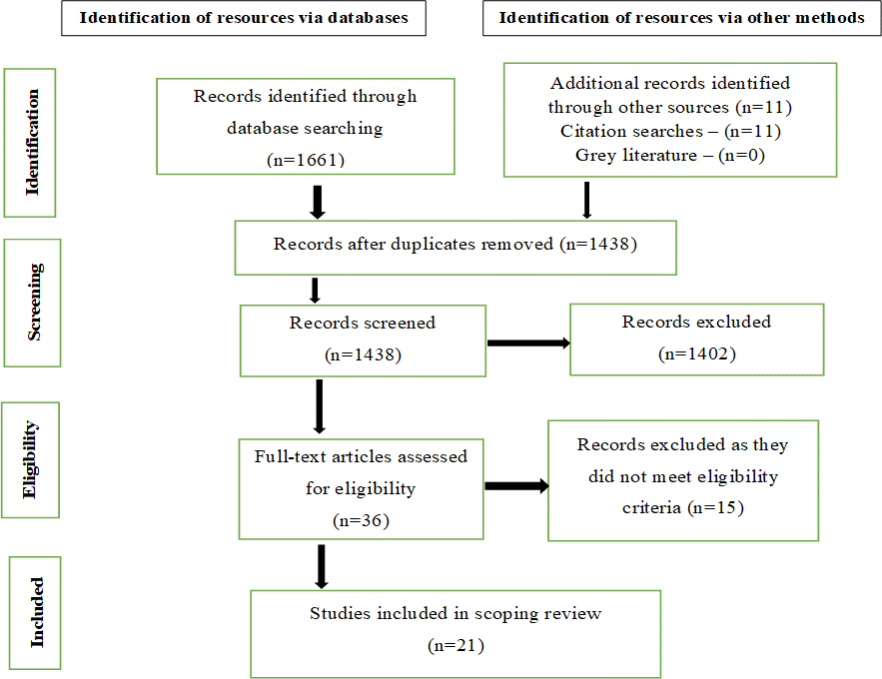
PRISMA flowchart. PRISMA: Preferred Reporting Items for Systematic Reviews and Meta-Analyses.

The database search identified 1683 articles, of which 245 (14.56%) were duplicates, so we screened the titles and abstracts of 1438 (85.44%) articles. A total of 36 studies were evaluated for eligibility, and 21 studies were included in the review. Most studies that remained included parents or caregivers as participants (16/21, 76%), while some studies examined children specifically (5/21, 24%). Figure 1 provides an overview of the study selection process and the reasons for the exclusions of full-text articles.

### Study Characteristics

All 21 articles included were published in peer-reviewed journals. The review included 4 (19%) studies conducted in China [17-20], 4 (19%) in the United States [21-24], 3 (14%) in India [25-27], 2 (10%) in the United Kingdom [28,29], 2 (10%) in Canada [30,31], 2 (10%) in Pakistan [32,33], 1 (5%) in Korea [34], 1 (5%) in the Netherlands [35], 1 (5%) in Israel [36], and 1 (5%) in Iran [37]. The sample size of the studies ranged from 59 to 6334. The studies identified various target populations, including children and adolescents aged between 0 and 19 years. Diverse interventions such as teaching, training, workshops, technology-based training, storybooks, educational book–based training, and model-based training were identified. The target population included parents, caregivers, or family members, as well as children and adolescents with ages ranging from 0 to 19 years. Table 1 provides a detailed overview of the study characteristics.

### Interventions Targeting Parents, Caregivers, and Family

Parents or caregivers supervise the activities of young children at home and are accountable for their environment. Hence, it is critical to assess their knowledge, attitudes, and practices regarding unintentional injuries and plan and implement effective interventions.

Parents or caregivers were the focus of intervention in 17 of 21 (81%) articles presented in this review. Caregivers of children below the age of 3 years were addressed in 8 of 17 (47%) articles [17,19,21,23,28,33-35], which focused on the knowledge of parents on vehicle safety, home safety, self-efficacy behaviors, and skill in first aid measures. Of the 17 articles, 6 (35%) highlighted interventions for parents of children aged less than 5 years [22,26,29,30,32,37], which assessed the levels of supervision and attention toward their children, and knowledge, practice, and safety behavior of parents regarding unintentional injuries. Two articles of 17 (12%) focused on caregivers of children between the ages of 3 and 6 years [18,31], and 1 article (6%) reported an intervention among caregivers of children younger than 12 years [24], which assessed the safety knowledge and safety actions of parents/caregivers, knowledge about hazards, injury risk behavior, and attitude toward injury prevention.

### Interventions Targeting Children as Change Agents

Injury prevention strategies, especially for individual-level factors, may be implemented using different learning approaches and theories. Approaches such as demonstrations, role-play, and simulation at the school level may help children understand how injury prevention interventions could work. Children learn about a health subject and share that information with their parents or the community, encouraging parents and others to enhance their knowledge and engage in health-promoting behaviors.

Of 21 articles, 3 (14%) focused on children of different age groups, evaluating the effectiveness of interventions to prevent unintentional injuries. Children in the third and fourth grades were assessed for their knowledge on the prevention of drowning, perceived vulnerability, and simulated behavior in and around the water [20]. Incidences and types of unintentional injuries were assessed among children in classes 5 to 7 [27]. In another study, through a child-to-child approach [25], adolescent children taught younger siblings and other family members regarding the prevention of unintentional injuries, whereby the knowledge of participants and the practices of the family were assessed.

### Effectiveness of Educational Interventions

Education provides individuals with the necessary knowledge and awareness of the benefits and risks associated with certain behaviors. Education stands as a powerful catalyst for change, enabling people to acquire skills, information, and perspectives that shape their attitudes and perceptions.

This review found that 5 of 21 articles (24%) reported various educational interventions as a single strategy or in combination with other modalities to prevent unintentional injuries in children. RISE UP is a tailored parenting program to promote nurturing, child development, resiliency, social connection, and support to prevent unintentional injuries, which showed a decrease in parenting injury risk scores and promoted positive parenting behaviors [22].

Safety education prepares children for specific situations, whereas risk education prepares them for unanticipated or unknown situations, where they may have had no specific instruction or learning opportunity. The child-to-child approach [25] is one such method wherein the older adolescents of the family were trained in first aid and cardiopulmonary resuscitation, road safety, traffic rules, injury prevention, and immediate care. As a next step, they were made to transfer the information to their younger siblings and other family members. In another study, a comprehensive pictorial child safety and injury prevention module, as part of a school-based intervention to prevent injuries and promote child safety, was evaluated [27]. A significant reduction in the incidence of injuries in younger children and improvement in knowledge and practice regarding injury prevention were noted.

A multifaceted intervention with components such as a youth leadership program for adolescents, workshops in well-baby clinics and preschools, home visits, and a media campaign showed a greater reduction in emergency room visits in children between 0 and 3 years of age, as well as reduced hospital admission due to burns and falls injury [36]. A significant reduction in injury hazards was identified in a group that received tutorials when compared to an educational pamphlet [32].

### Effectiveness of Home Safety Interventions

Home is a place where children spend lots of time during their younger days. It is the responsibility of the caregivers to ensure a safe place for the kids to learn, play, and explore. Home safety interventions are implemented to reduce the risk of accidents, injuries, and other hazards within households. In total, 4 of 21 articles (19%) reported on the effectiveness of home safety interventions.

Implementation of home-based injury prevention programs through the “Watchful Parents, Safe Children” video, “Safe Home, Safe Child” video, and a poster followed by a structured discussion with mothers and home visits showed a significant decrease in time during which the children were unsupervised, higher levels of supervision, an increase in attention to the child, a significant reduction in the injury pattern in children, and improved mean scores of caregivers’ home safety practices [26,30].

Implementation of innovative teaching strategies and playful approaches aimed at children can have a long-lasting effect in reducing unintentional injuries. A storybook, “Careful Puppy Saves the Day,” with information on different hazards in and around the home, read by mothers with their children beside them, was incorporated as a change agent. Significant changes were noted in the form of better identification of injury hazards, fewer risky behaviors, and a more comprehensive explanation of safety by the children [31]. Furthermore, Stay One Step Ahead, a multievidence-based intervention for families, incorporated monthly safety messages through quizzes, posters, and flyers; organized home safety activities; and educational safety weeks for families on common injuries. The findings showed a significant improvement in the storage of poisons out of children’s reach and improved use of home safety measures [28].

### Technology-Based Interventions

Technology-based interventions can provide individualized and tailored interfaces enriched with interactive elements [38], which can be accessed anytime and anywhere. This review identified 9 articles that dealt with the effectiveness of technology-based interventions to prevent various forms of unintentional injuries in children.

A video-based DVD on child safety seat installation and use [23]; a web-based, E-health4Uth module along with personal counseling on the prevention of falls, poisoning, drowning, and burns [35]; a testimonial-based video intervention in reducing drowning risk among school-aged children [20]; and an app-based parenting education intervention to prevent unintentional injury [18] are a few of the studies that used technology as a medium to disseminate the information to children and caregivers. The results of the studies show an improvement in their respective areas of assessment, which include the ability of caregivers to rightly install the car seat, less unsafe injury-prone behaviors, and children’s safety knowledge of drowning risk.

SafeTea is a community-based intervention that incorporates video clips, posters, leaflets, and charts to educate parents regarding the risk factors for hot drink scalds and first aid for burns [29]. Choi and Ahn [34] conducted a mobile-based parental education program to prevent unintentional injuries in children, and McKenzie et al [24] studied the effectiveness of the Make Safe Happen App, which helped caregivers keep their homes safe and also provided links to purchase home safety products from Amazon.com. Two studies [17,19] used WeChat accounts for social-network–based health education to enhance the knowledge, skill, and beliefs of parents about unintentional injuries.

### Interventions Based on Models and Theories

A theory is a comprehensive framework for explaining and predicting phenomena, while a model is a focused and practical tool that represents parts of a theory, helping to visualize or analyze specific aspects. We found that 3 of 21 articles (14%) reported on the effectiveness of interventions based on theories and models.

Two studies [33,37] evaluated the effect of training programs on mothers’ knowledge regarding the prevention of unintentional injuries in children based on the Health Belief Model. Wang et al [21] assessed the effectiveness of an intervention grounded in social cognitive theory. The outcome of the reviewed article showed an improvement in the mothers’ knowledge and practice, a decrease in the number of injuries, a significant difference in the Health Belief Model constructs, and fewer safety problems. Health education models can be used when planning educational programs at the individual or community levels.

## Discussion

### Principal Findings

The study aimed to answer the following scoping review question: what is known globally from the existing research literature about interventions to prevent unintentional injuries in children?

This review highlights the different areas of unintentional injuries, the interventions to prevent them, the outcomes of the interventions, and the diverse population on whom an intervention is being implemented. It provides an overview of how the interventions have been conceptualized and applied with the use of various data collection instruments measured at different timelines and their outcome measures. Of 21 studies considered for review, 16 (76%) studies were RCTs, 4 (19%) articles were non-RCTs, and 1 (5%) article had a mixed method design. Due to the heterogeneous nature of the included studies, the level of evidence of this review is considered as moderate.

### Interventions Targeting Parents, Caregivers, and Family

In this review, 17 articles described interventions focusing on caregivers or parents to prevent unintentional injuries in children. Parents’ or caregivers’ involvement in educational interventions has been shown to improve safety knowledge and behaviors. In addition, the distribution of safety supplies to keep the home safe has improved the safety-related behavior of parents. Similar evidence was reported by the authors of a review [39] that interventions targeting parents are effective in reducing child injuries and that parenting interventions appeared to have a greater effect on home safety practices and the reduction of hazards at home. The findings of this review suggested that the beneficial effects were attributable to interventions targeting changes in parents’ knowledge, attitude, and behavior.

### Interventions Targeting Children as Change Agents

Three articles highlighted the interventions for preventing unintentional injuries in diverse modalities focusing on children of different age groups. The analysis showed improved knowledge and safety practices and a decrease in the incidence of injuries. However, there are fewer studies focused on children, who need to be considered further. There is a need to integrate appropriate and well-designed programs, especially focusing on children, such as school-based injury prevention modules, adolescent-targeted interventions, and simulation-based training. The cost of preventing unintentional injuries is much lower than the cost of treating their direct and indirect consequences [40]. In resource-limited settings, children can act as change agents to bring about change in their homes and neighborhoods. Future research on children should engage parents and teachers to develop, implement, and evaluate a more comprehensive and integrated intervention to prevent unintentional injuries.

### Effectiveness of Educational Interventions

Five articles in this review addressed the effectiveness of educational interventions for both parents/caregivers and children. Educational interventions targeting changes in the behavior of parents and a hazard-free home environment were proven effective in reducing the incidence of unintentional injuries at home. The results of the studies demonstrate a positive outcome and ensure their feasibility of being implemented in low-income and resource-limited settings. Despite the high incidence of unintentional injuries among children in low- and middle-income countries, few interventional studies have been conducted in these countries. Multimodal educational interventions play a crucial role in sensitizing and creating awareness among children and caregivers to prevent unintentional injuries and injury hazards.

### Effectiveness of Home Safety Interventions

Four articles reported on home safety interventions to prevent unintentional injuries. The analysis showed improved home safety practices, an increased level of supervision, and an environment free from injury hazards. Greater reductions in injury rates were found for interventions delivered in the home; however, there was a lack of evidence that home safety interventions reduced rates of thermal injuries or poisoning [41]. It is particularly important that children in low-income families have close supervision, as the caregivers may have difficulty acquiring safety materials, and the presence of poor housing quality can impose an additional risk on children. Hence, the implementation of low-cost and locally available measures in an accessible and efficient manner could prove beneficial for both children and their caregivers to reduce the risk of injuries within the community context. Failure to invest in programs for preventing injuries among children will further increase the number of dependents in future generations and negatively impact society. Policymakers need to be involved in the evaluation and implementation of policies related to injury prevention, especially in resource-limited countries.

### Technology-Based Interventions

Nine articles addressed the effectiveness of technology-based interventions in the prevention of unintentional injuries. Studies using interventions through mobile apps, web-based education, and social online health education provided an impactful result among the participants who used them effectively on a timely basis. Information and communication technologies can accelerate progress toward the achievement of the United Nations Sustainable Development Goals [42]. The use of technology-based interventions and an online platform can be adapted to deliver preventive health care information for large-scale community users. Nevertheless, the digital mode of interventions and app-based programs play a significant role in covering the large and diverse range of participants to create awareness, instill knowledge, improve their practice, and bring about a positive change in their behaviors. However, such interventions have drawbacks in low- and middle-income countries with a lack of resources and a lack of participant awareness of different gadgets.

### Interventions Based on Models and Theories

This review highlighted 3 articles that used models and theories as a basis for interventions to prevent injuries. The Health Belief Model is a theoretical model used to guide health promotion and disease prevention programs, which explains and predicts changes in health behaviors [43]. Accordingly, the previously mentioned studies proved useful in applying a similar approach to evaluate the impact of interventions and thus bring about change in the practice and behavior of parents.

### Strengths and Limitations

A strength of this review was that a comprehensive literature search of articles published from 2013 to 2023 was conducted using different databases that covered studies focusing on interventions to prevent unintentional injuries in children. A limitation of our review was that only articles published in the English language were included. Additionally, the present study area is an active field of research, so it is important to note that this scoping review is a snapshot at a particular point in time.

### Conclusion

The articles included in this review addressed interventions of different magnitudes, data measurements taken at varying time points, and participants of a diverse nature. Cost-effective, accessible, and multifaceted interventions represent effective strategies to prevent unintentional injuries in children. Further studies in the form of online social networks and app-based interventions could be considered to reach out to the larger population with long-term outcome measurements.

## Supplementary material

10.2196/67877 Checklist 1 PRISMA-ScR checklist.

## Abbreviations

PRISMA-ScR: Preferred Reporting Items for Systematic Reviews and Meta-Analyses extension for Scoping Reviews
RCT: randomized controlled trial

## References

[1] Morrongiello BA (2005). Caregiver supervision and child-injury risk: I. Issues in defining and measuring supervision; II. Findings and directions for future research. J Pediatr Psychol.

[2] Peden M (2008). World report on child injury prevention. World Health Organization.

[3] Sengoelge M, Leithaus M, Braubach M, Laflamme L (2019). Are there changes in inequalities in injuries? A review of evidence in the WHO European Region. Int J Environ Res Public Health.

[4] Kumar M, Pathak VK, Tripathi S, Upadhyay A, Singh VV, Lahariya C (2023). Burden of childhood injuries in India and possible public health interventions: a systematic review. Indian J Community Med.

[5] Pinheiro PS (2006). World report on violence against children. https://violenceagainstchildren.un.org/sites/violenceagainstchildren.un.org/files/document_files/world_report_on_violence_against_children.pdf.

[6] Sethi D (2008). European report on child injury prevention. https://iris.who.int/bitstream/handle/10665/326500/9789289042956-eng.pdf.

[7] Schwebel DC, Gaines J (2007). Pediatric unintentional injury: behavioral risk factors and implications for prevention. J Dev Behav Pediatr.

[8] Flavin MP, Dostaler SM, Simpson K, Brison RJ, Pickett W (2006). Stages of development and injury patterns in the early years: a population-based analysis. BMC Public Health.

[9] Sheriff A, Rahim A, Lailabi MP, Gopi J (2011). Unintentional injuries among children admitted in a tertiary care hospital in North Kerala. Indian J Public Health.

[10] Bhuvaneswari N, Prasuna JG, Goel MK, Rasania SK (2018). An epidemiological study on home injuries among children of 0-14 years in South Delhi. Indian J Public Health.

[11] Morrongiello BA, Schell SL (2010). Child injury: the role of supervision in prevention. Am J Lifestyle Med.

[12] Ablewhite J, Kendrick D, Watson M, Shaw I (2015). Maternal perceptions of supervision in pre-school-aged children: a qualitative approach to understanding differences between families living in affluent and disadvantaged areas. Prim Health Care Res Dev.

[13] Harris VA, Rochette LM, Smith GA (2011). Pediatric injuries attributable to falls from windows in the United States in 1990-2008. Pediatrics.

[14] Chandran A, Khan UR, Zia N (2013). Disseminating childhood home injury risk reduction information in Pakistan: results from a community-based pilot study. Int J Environ Res Public Health.

[15] Arksey H, O’Malley L (2005). Scoping studies: towards a methodological framework. Int J Soc Res Methodol.

[16] Tricco AC, Lillie E, Zarin W (2018). PRISMA Extension for Scoping Reviews (PRISMA-ScR): checklist and explanation. Ann Intern Med.

[17] Feng Y, Li X, Ma X (2022). Using online social networks to provide a parental health-education intervention for preventing unintentional injuries among children aged 0-3 years: a randomized controlled trial and social network analysis in Shanghai, China. Front Public Health.

[18] Ning P, Cheng P, Schwebel DC (2019). An app-based intervention for caregivers to prevent unintentional injury among preschoolers: cluster randomized controlled trial. JMIR mHealth uHealth.

[19] Feng Y, Ma X, Zhang Q (2022). Effectiveness of WeChat-group-based parental health education in preventing unintentional injuries among children aged 0-3: randomized controlled trial in Shanghai. BMC Public Health.

[20] Shen J, Pang S, Schwebel DC (2016). Evaluation of a drowning prevention program based on testimonial videos: a randomized controlled trial. J Pediatr Psychol.

[21] Wang Y, Gielen AC, Magder LS, Hager ER, Black MM (2018). A randomised safety promotion intervention trial among low-income families with toddlers. Inj Prev.

[22] Weaver NL, Weaver TL, Loux T, Jupka KA, Lew D, Sallee H (2019). The impact of RISE Up! in promoting positive parenting and safety behaviors of parents with young children. Child Youth Serv Rev.

[23] Swartz L, Glang A, Schwebel DC, GeigerWolfe EG, Gau J, Schroeder S (2013). Keeping baby safe: a randomized trial of a parent training program for infant and toddler motor vehicle injury prevention. Accid Anal Prev.

[24] McKenzie LB, Roberts KJ, Collins CL, Clark RM, Smith KC, Manganello J (2019). Maternal knowledge, attitudes, and behavioral intention after exposure to injury prevention recommendations in the news media. J Health Commun.

[25] Banerjee B, Banerjee R, Ingle GK, Mishra P, Sharma N, Garg S (2021). Effectiveness of child-to-child approach in preventing unintentional childhood injuries and their consequences: a non-randomized cluster-controlled trial. Indian Pediatr.

[26] George A, Renu G, Shetty S (2021). Effect of a home safety supervisory program on occurrence of childhood injuries: a cluster randomized controlled trial. Indian Pediatr.

[27] Holla R, Darshan BB, Unnikrishnan B (2021). Effectiveness of school-based interventions in reducing unintentional childhood injuries: a cluster randomized trial. Indian Pediatr.

[28] Taylor MJ, Orton E, Patel T (2023). Effectiveness of systematically delivered evidence-based home safety promotion to improve child home safety practices: a controlled before-and-after study. Inj Prev.

[29] Cowley LE, Bennett CV, Brown I, Emond A, Kemp AM (2021). Mixed-methods process evaluation of SafeTea: a multimedia campaign to prevent hot drink scalds in young children and promote burn first aid. Inj Prev.

[30] Morrongiello BA, Zdzieborski D, Sandomierski M, Munroe K (2013). Results of a randomized controlled trial assessing the efficacy of the Supervising for Home Safety program: impact on mothers’ supervision practices. Accid Anal Prev.

[31] Morrongiello BA, Marquis AR, Cox A (2021). A RCT testing if a storybook can teach children about home safety. J Pediatr Psychol.

[32] Khan UR, Ali A, Khudadad U (2023). Follow-up household assessment for child unintentional injuries two years after the intervention: a community-based study from Karachi, Pakistan. Injury.

[33] Tajiki I, Vizeshfar F, Keshtkaran Z (2022). The effect of training program based on health belief model on burn prevention knowledge in mothers of children aged to 1-3 years: a randomized controlled. Burns.

[34] Choi Y, Ahn HY (2021). Developing and evaluating a mobile-based parental education program for preventing unintentional injuries in early childhood: a randomized controlled trial. Asian Nurs Res (Korean Soc Nurs Sci).

[35] van Beelen MEJ, Beirens TMJ, den Hertog P, van Beeck EF, Raat H (2014). Effectiveness of web-based tailored advice on parents’ child safety behaviors: randomized controlled trial. J Med Internet Res.

[36] Myers V, Malkin G, Nir N, Orr D, Baron-Epel O (2022). Evaluation of an intervention to reduce child injury in Bedouin communities in Southern Israel. Inj Prev.

[37] Cheraghi P, Poorolajal J, Hazavehi SMM, Rezapur-Shahkolai F (2014). Effect of educating mothers on injury prevention among children aged <5 years using the Health Belief Model: a randomized controlled trial. Public Health (Fairfax).

[38] Mouton A, Cloes M (2013). Web-based interventions to promote physical activity by older adults: promising perspectives for a public health challenge. Arch Public Health.

[39] Kendrick D, Mulvaney CA, Ye L, Stevens T, Mytton JA, Stewart-Brown S (2013). Parenting interventions for the prevention of unintentional injuries in childhood. Cochrane Database Syst Rev.

[40] Salam RA, Arshad A, Das JK (2016). Interventions to prevent unintentional injuries among adolescents: a systematic review and meta-analysis. J Adolesc Health.

[41] Kendrick D, Young B, Mason-Jones AJ (2013). Home safety education and provision of safety equipment for injury prevention (review). Evid Based Child Health.

[42] Digital technologies to achieve the UN SDGs. ITU.

[43] Alyafei A, Easton-Carr R (2024). The Health Belief Model of Behavior Change.

